# Psychosocial determinants of adolescent romantic relationship in Malaysia: Social media use, pornography surfing, sexual and reproductive health knowledge, and depression

**DOI:** 10.1371/journal.pone.0295933

**Published:** 2023-12-20

**Authors:** Muhammad Ikhwan Mud Shukri, Anisah Baharom

**Affiliations:** Department of Community Health, Faculty of Medicine and Health Sciences, Universiti Putra Malaysia, Selangor, Malaysia; Universidade do Estado do Rio de Janeiro, BRAZIL

## Abstract

It is socially natural that adolescents engage in romantic relationship. However, there are negative health implications when they are not properly monitored and guided. The engagement in unsafe sexual practices has been reported to cause various adverse health and social implications affecting Malaysian adolescents. To date, why adolescents engage in romantic relationship has remained understudied. Prior studies on adolescent romantic relationship mainly focused on the western context. Hence, the reported findings are deemed not applicable for the Malaysian population. There are insufficient data on the prevalence and determinants of adolescent romantic relationship within the Malaysian context. Thus, the current study aimed to identify the prevalence and determinants of romantic relationship among adolescents in Seremban, Negeri Sembilan, Malaysia. Adopting a cross-sectional research design, this study’s self-administered questionnaire survey, which was conducted from 25 May 2021 to 5 June 2021 in residential areas that were part of the “Healthy Community Empowers the Nation” programme (KOSPEN), involved 396 adolescents who fulfilled the study’s selection criteria. In terms of age, the respondents were of between 14 and 19 years old, with median age (IQR) of 19 (2). Adolescent romantic relationship in this study recorded prevalence of 24.1%, which was comparatively lower than that of the western countries. The results further revealed social media use (AOR: 2.162, 95% CI: 1.202–3.888, p = 0.01), pornography website surfing (AOR 2.748, 95% CI: 1.517–4.977, p = 0.001), poor SRH knowledge (AOR: 3.885, 95% CI: 2.144–7.040, p < 0.001), and depression (AOR: 2.830, 95% CI: 1.323–6.055, p = 0.007) as significant determinants of adolescent romantic relationship. Thus, this study demonstrated the significant role of social media use, pornography website surfing, SRH knowledge, and depression on adolescent romantic relationship. Further longitudinal studies to investigate the temporal relationships between depression and romantic relationship are recommended. The highly significant association between SRH knowledge and romantic relationship in this study suggests that strategies on improving the level of SRH knowledge among adolescents should be prioritised.

## Introduction

According to the World Health Organisation (WHO), adolescents are individuals of the age of between 10 and 19 years old. They make up 16% of the global population, amounting to 1.2 billion people [1]. As compared to other age groups, the mortality rate of adolescents is generally lower. In 2012 alone, about 1.3 million adolescents died worldwide, with road injury, HIV, and suicide as some of the leading causes of death. Since 2000, the number of HIV-related deaths among adolescents has tripled or more, making it the second cause of mortality worldwide [2].

Improving the health and well-being for all, including adolescents, is part of SDG 3. As part of the global efforts to end HIV epidemic by 2030, the primary prevention of HIV infection is emphasised, especially among adolescents as population at risk to be infected [3]. Safe practices in dating and romantic relationship among adolescents can prevent unsafe sexual relationship, which subsequently leads to the transmission of HIV and sexually transmitted diseases.

The main feature of social development in adolescence lies in the initiation of romantic relationship. It is a new and mutual experience that is characterised by intense emotions or feelings of love, passion, and sexuality [4]. Different cultures view behaviours and commitments in romantic relationships differently, resulting in the complexities of defining romantic and intimate relationship accurately [5]. Emotional connection and sexual behaviours typically characterise a romantic relationship that may or may not occur in the context of a relationship. Although the definition of romantic relationship includes the notion of erotic feelings, which distinguish romantic relationships from friendships, such relationship does not require erotic or sexual behaviours [6]. Romantic relationships are commonly initiated in early adolescence and then progress into more committed, emotional, and sexual relationships in late adolescence.

In the United States (US), it was reported that about half of adolescents engaged in romantic relationship by the age of 15 [7]. There are different theories on the biological, psychological, psychosocial, and cognitive developments in adolescence. Most girls experience physical growth and pubertal maturation as early as eight years old; girls generally experience pubertal changes earlier than boys. Two children of the same or different gender first interact and form a friendship, which grows to dating and romantic relationship in their adolescence [8]. At the age of 9, children can differentiate the conceptions of romantic relationships and friendships; they uniquely identify romantic relationship as longing, physical attraction, and commitment [9]. On the other hand, friendships of different gender are viewed as less intimate than friendships of same gender [10], implying a continuum of intimacy from friendships of different gender at the lower end to romantic relationships at the higher end [6].

Dating activities progress from hanging out in mixed-gender group and forming friendships to dating [11]. Dating is casual and typically takes place in peer group. A relationship may form into a dyadic romantic relationship when dating activities take place [12]. The engagement in romantic relationship is considered as a joyous and thrilling experience for adolescents [13]. Every individual displays different tendency to engage in a romantic relationship. This is influenced by different determinants of romantic relationship initiation, such as demographic characteristics, personality traits, substance use, pornographic website surfing, and attachment style [14–19].

In a survey study, male adolescents were found to be more inclined to form romantic relationship, with secure attachment style identified as contributing factor [20]. More recent studies also showed elder adolescents, alcoholic drinkers, illegal drug abusers and depressed adolescents were among the significant risk factors [17, 18, 21].

For the past few years, internet sites and online social media have become one of the avenues for adolescents to seek romantic relationship. Almost 92% of adolescents are accessing internet daily [22]. In Malaysia, six out of seven (85.7%) adolescents are active internet users [23]. They are using internet sites and mobile phone dating apps such as Facebook, Instagram Twitter, Tinder and Kik to build romantic relationship [24]. A 9-year longitudinal study involving more than 2000 adolescent student in Taiwan showed that online social networking significantly increases the rates of first romantic relationship by 1.657 times whereas surfing pornographic websites increases the odds of a sexual debut in adolescents by 53.3% [14].

Sexual and reproductive health (SRH) knowledge is imperative among adolescents, as it serves as the foundation of good sexual- and health-related behaviours. Despite that, adolescents have inadequate access to SRH knowledge [25]. The lack of studies on SRH knowledge in regards to adolescent romantic relationship has revealed a gap in literature. Focusing on risky sexual behaviour, a proxy study adopted a cross-sectional research design and reported the significant relationship between students who were not fully exposed to the interventional health educational programme (indicated SRH knowledge) and risky sexual behaviour (AOR: 1.5, 95% CI: 1.1–1.8, p = 0.008) based on a sample of 1,364 high school students in West Papua, Indonesia [26].

Parental divorce has been identified as a predictor of adolescent romantic relationship. Following separation process, adolescents are more likely to pull away from parents and look for emotional support [27]. The desire for closeness to a partner may result from the divorce [28]. A recent study found adolescents from divorced families are at higher risk of developing romantic relationship than those from intact family [15]. Similarly, recent literature demonstrated adolescents who were more intimate in their friendship were more likely to be in romantic relationship compared to those who possessed more companionship characteristics in friendship quality [19]. Additionally, poor parental monitoring has been identified as risk factors for romantic relationship among adolescents [16]. In summary, the factors were found to be associated with romantic relationship among adolescents were 1) individual factors i.e., age, gender, alcohol intake, illegal drug use, social media use, pornography website surfing, SRH knowledge, and depression; and 2) parental factors i.e., parental marital status and parental monitoring; and 3) friendship quality.

With the engagement of adolescents in romantic relationship, positive (e.g., development of self-esteem and personal identity) and negative consequences are bound to occur gradually. Although positive consequences of romantic relationship in adolescents are essential in adolescent developmental task, it is crucial to prioritise efforts to deal with the negative consequences, such as mental health disorders, sexually transmitted diseases, and substance use.

Accordingly, there are several determinants on why adolescents start to have the intention to form romantic relationship. To the extent of our knowledge, there have been limited studies on adolescent romantic relationship within the Malaysian context. Addressing that, the current study aimed to identify the prevalence and determinants of adolescent romantic relationship in Malaysia.

## Methods

Adopting a cross-sectional research design, the current study targeted adolescents living in residential areas that were part of the “Healthy Community Empowers the Nation” programme (*Komuniti Sihat Pembina Negara—*KOSPEN) in Seremban, Negeri Sembilan, Malaysia. The self-administered questionnaire, which was developed in English language, was constructed based on prior studies. Overall, there were five main sections. The first section, which consisted of 18 questions, focused on factors like age, gender, alcohol intake, illegal drug use, social media use, pornography website surfing, and SRH knowledge. SRH knowledge in this study was measured using 12 items. For each item, respondents were required to select one option from the following: “True”, “False”, or “Don’t Know”. The number of correct answers for each item in this study represented SRH knowledge score [29]. With the median split method, the cut-off point of eight was used to categorise the level of SRH knowledge into “poor” and “good”.

The second section, which consisted of nine questions, focused on parental factors like parental marital status and parental monitoring. Parental Monitoring Scale [30], which consisted of eight items with a five-point scale, was used to measure the level of parental monitoring in this study. A higher mean score indicated a higher perceived level of parental monitoring. With the median split method, the cut-off point of 33 was used to categorise the level of parental monitoring into “low” and “high”.

The third section focused on friendship quality, which incorporated Parker and Asher’s [31] Friendship Quality Questionnaire. Three relevant domains [19] were selected to measure the friendship quality score in relation to romantic relationship: (1) companionship and recreation; (2) intimate disclosure and conflict; (3) betrayal. A five-point scale was used, where each respondent answered the questions with a specific friend in mind. The score for each domain was measured by considering the average total score per unit item.

The fourth section focused on depression, which incorporated Beck Depression Inventory-II (BDI-II) [32]. BDI-II was selected because it demonstrated appropriate properties to measure depression among school students with high sensitivity in detecting depression within the Malaysian context based on a 2004 study conducted by Wan Mahmud [33]. The 21^st^ question on women’s sexual libido in the original BDI-II was not included in the current study’s questionnaire, resulting in the use of 20-item BDI-II instrument instead. With a list of four statements arranged in increasing severity about depression symptoms, each item was scored on a scale of “0” to “3”. The cut-off point of 10 [33] was used to categorise respondents into “having depression” and “not having depression”. The fifth and final section, which consisted of six questions on romantic relationship, focused on the level of adolescence social relationship and the definition of romantic relationship (based on elements of intimacy, emotional closeness, and erotic feeling) [12, 34].

The developed questionnaire in English language was later translated into Malay language by two public health physicians who are bilingual (English language and Malay language). Following that, the questionnaire in Malay was back-translated by two primary care physicians who are well-versed in both languages.

Following that, a pre-test, which involved 30 adolescents from three other KOSPEN residential areas, was conducted to assess face validity of the questionnaire. These adolescents were excluded from the actual data collection. Respondents of the pre-test were required to comment on the questionnaire structure and sentences. The results revealed no need for modification. In addition, three public health physicians were appointed to assess content validity of the questionnaire. Certain modifications were done based on their recommendations. All items of 11 independent variables and a dependent variable recorded content validity ratio (CVR) value of 1. Based on these obtained values, all items were deemed essential. Thus, all items were retained. Cronbach’s coefficient and Kuder-Richardson 20 [35] were used to assess the reliability of the questionnaire. Overall, the internal consistency values ranged from 0.658 to 0.890.

Random cluster sampling strategy was adopted in this study. The estimated population of adolescents aged 14 to 19 years old in 25 KOSPEN residential areas in 2021, which excluded three other residential areas used in pre-test, was 503. The lists of adolescents aged 14 to 19 years old were gathered from the designated head of each selected residential area. The estimated average population of adolescents aged 14 to 19 years old in each residential area was 20 (N). Considering the required sample size (n = 396), the number of clusters (residential areas) equals to 20 clusters (n/N = 396/20). With that, this study randomly selected 20 clusters of residential areas from a total of 25 clusters prior to the random selection of Malaysian adolescents in the age group of between 14 and 19 years old. Data collection using a validated, self-administered questionnaire survey was conducted from 25 May 2021 to 5 June 2021. Questionnaires were distributed to 396 respondents through the heads of the selected residential areas and were collected within a period of one week. Informed consents were obtained from all participating students and their parents or legal guardians using official written documents.

IBM SPSS (version 25) was used for data entry and analysis to minimise data entry error. Data distribution was evaluated using the Shapiro-Wilk test, Kolmogorov-Smirnov test, and assessment of kurtosis and skewness. Chi-squared test was also employed to examine the significance of the relationships involving the categorical independent and dependent variables. In addition, multiple logistic regression model was utilised to control the effects of possible confounders at 0.05 level of significance [36]. Study protocol and processes were approved by the Ethics Committee for Research Involving Human Subjects of Universiti Putra Malaysia (ID number: JKEUPM-2021-190). Informed consent was obtained from all study participants.

## Results

Out of 396 respondents selected, 345 respondents agreed to participate in the current study. The recorded response rate of 87.1% was deemed good. The prevalence of adolescent romantic relationship in this study recorded 24.1%. Table 1 presents the descriptive statistics of the studied determinants and adolescent romantic relationship among respondents in this study. In terms of age, the respondents were of between 14 and 19 years old, with median age (IQR) of 19 (2). Most of the respondents in this study were late adolescents (75.4%), specifically of the age of between 17 and 19 years old. About 82.6% of the total respondents were female adolescents. Some of these respondents revealed to have drunk alcohol (3.8%) and used illegal drugs (1.7%). Besides that, 28.7% of the total respondents reported to have used social media to seek romantic relationship. About 27.0% revealed to have surfed pornography website. Only a few respondents were from family of divorced parents (5.8%). Most of the respondents had high level of parental monitoring (53.9%) and expressed to have experienced depression (79.4%).

**Table 1:** Descriptive statistics of the studied determinants and adolescent romantic relationship.

Variables	Frequency (N = 345)	Percentage (%)
**Individual Factors**		
**Age (years)**		
** 14–16 (middle-aged adolescents)**	85	24.6
** 17–19 (late adolescents)**	260	75.4
**Gender**		
** Male**	60	17.4
** Female**	285	82.6
**Alcohol Intake**		
** Yes**	13	3.8
** No**	332	96.2
**Illegal Drug Use**		
** Yes**	6	1.7
** No**	339	98.3
**Social Media Use**		
** Yes**	99	28.7
** No**	246	71.3
**Pornography Website Surfing**		
** Yes**	93	27.0
** No**	252	73.0
**Sexual and Reproductive Health Knowledge**		
** Good (> 8)**	174	50.4
** Poor (≤ 8)**	171	49.6
**Depression**		
** Yes**	274	79.4
** No**	71	20.6
**Parental Factors**		
**Parental Marital Status**		
** Divorced**	20	5.8
** Others**	325	94.2
**Parental Monitoring**		
** High (> 33)**	186	53.9
** Low (≤ 33)**	159	46.1
**Friendship Quality Score**	**Median (IQR)**	
** Companionship and Recreation**	20.00 (5.00)	
** Intimate Exchange**	24.00 (8.00)	
** Conflict and Betrayal**	13.00 (5.00)	
**Adolescent Romantic Relationship**		
** Yes**	83	24.1
** No**		
** Same-Gender Friendship**	91	26.4
** Opposite-Gender Friendship**	155	44.9
** Dating Relationship**	16	4.6

Meanwhile, the results on the relationships involving the studied determinants and romantic relationship are summarised in Table 2. In chi-squared test, p-value of less than 0.05 indicates a statistically significant relationship between the studied variables [36]. The obtained results demonstrated the significant relationships of social media use, pornography website surfing, poor SRH knowledge, and depression with adolescent romantic relationship.

**Table 2:** Results of bivariate analysis on the relationships involving the studied determinants and adolescent romantic relationship.

Determinants	Romantic Relationship		*χ* ^2^	df	p-value
	Yes n = 83 (%)	No n = 262 (%)			
**Age (years)**					
** 14–16**	21 (24.7)	64 (75.3)	0.026	1	0.872
** 17–19**	62 (23.8)	198 (76.2)			
**Gender**					
** Male**	20 (33.3)	40 (66.7)	3.420	1	0.064
** Female**	63 (22.1)	222 (77.9)			
**Alcohol Intake**					
** Yes**	3 (23.1)	10 (76.9)	0.007	1	0.993
** No**	80 (24.1)	252 (75.9)			
**Illegal Drug Use**					
** Yes**	3 (50.0)	3 (50.0)	2.249	1	0.134
** No**	80 (23.6)	259 (76.4)			
**Social Media Use**					
** Yes**	32 (32.3)	67 (67.7)	5.191	1	0.023*
** No**	51 (20.7)	195 (79.3)			
**Pornography Website Surfing**					
** Yes**	33 (35.5)	60 (64.5)	9.098	1	0.003*
** No**	50 (19.8)	202 (80.2)			
**Sexual and Reproductive Health Knowledge**					
** Good**	29 (16.7)	145 (83.3)	10.497	1	0.001*
** Poor**	54 (31.6)	117 (68.4)			
**Depression**					
** Yes**	73 (26.6)	201 (73.4)	4.867	1	**0.027** *
** No**	10 (14.1)	61 (85.9)			
**Parental Marital Status**					
** Divorced**	6 (30.0)	14 (70.0)	–	1	0.590**
** Others**	77 (23.7)	248 (76.3)			
**Parental Monitoring**					
** High**	37 (19.9)	149 (80.1)	3.833	1	0.050
** Low**	46 (28.9)	113 (71.1)			

*p < 0.05

**Fisher’s exact test

The obtained regression results in Table 3 demonstrated the statistical significance of social media use, pornography website surfing, SRH knowledge, and depression in the final multiple logistic regression model. After controlling for all other variables in the model, the odds of having romantic relationship among respondents who reported using social media use (AOR: 2.162, 95% CI: 1.202–3.888, p = 0.01), having to surf pornography website (AOR: 2.748, 95% CI: 1.517–4.977, p = 0.001), and having depression (AOR: 2.830, 95% CI: 1.323–6.055, p = 0.007) were twice higher than those who did not use social media, did not surf pornography website, and did not have depression. Meanwhile, the odd of having romantic relationship among respondents who demonstrated poor level of SRH knowledge was thrice higher than those with good level of SRH knowledge (AOR: 3.885, 95% CI: 2.144–7.040, p < 0.001). The results of Omnibus test further revealed statistical significance (Omnibus *χ*^2^ = 38.949, p < 0.001), which indicated that the model can distinguish between respondents with romantic relationship or otherwise. The model was explained by 10.7% (Cox and Snell R^2^) and 16.0% (Nagelkerke R^2^) of the variance in adolescent romantic relationship and correctly classified 74.2% of the cases (Classification Table). With the Receiver Operating Characteristic (ROC) curve, the final model significantly discriminated 72.3% of the cases (p < 0.001, CI: 0.667–0.779).

**Table 3:** Results of simple logistic regression and multiple logistic regression for predicting determinants of adolescent romantic relationship.

	Simple logistic regression					Multiple logistic regression				
Variables	B	S.E.	Wald	COR (95% CI)	p-value	B	S.E.	Wald	AOR (95% CI)	p-value
**Constant**						-3.320				
**Age (years)**										
** 14–16**	Ref									
** 17–19**	-0.047	0.291	0.026	0.954 (0.593–1.852	0.872					
**Gender**										
** Female**	Ref									
** Male**	0.566	0.309	3.364	1.762 (0.962–3.227)	0.067**					
**Alcohol Intake**										
** No**	Ref									
** Yes**	-0.057	0.671	0.007	0.945 (0.254–3.518)	0.933					
**Illegal Drug Use**										
** No**	Ref									
** Yes**	1.175	0.826	2.021	3.237 (0.641–16.356)	0.155**					
**Social Media Use**										
** No**	Ref					Ref				
** Yes**	0.602	0.266	5.114	1.826 (1.084–3.078)	**0.024** *	0.771	0.299	6.631	2.162 (1.202–3.888)	**0.010** *
**Pornography Website Surfing**										
** No**	Ref					Ref				
** Yes**	0.798	0.268	8.863	2.222 (1.314–3.759)	0.003*	1.011	0.303	11.131	2.748 (1.517–4.977)	**0.001** *
**SRH Knowledge**										
** Good**	Ref					Ref				
** Poor**	0.836	0.262	10.217	2.308 (1.382–3.854)	**0.001** *	1.357	0.303	20.016	3.885 (2.144–7.040)	**< 0.001** *
**Depression**										
** No**	Ref					Ref				
**Y es**	0.795	0.368	4.685	2.215 (1.078–4.553)	**0.030** *	1.040	0.388	7.184	2.830 (1.323–6.055)	**0.007** *
**Parental Marital Status**										
** Divorced**	Ref									
** Others**	0.322	0.505	0.407	1.380 (0.513–3.715)	0.523					
**Parental Monitoring**										
** Good**	Ref									
** Poor**	0.494	0.254	3.798	1.639 (0.997–2.695)	0.051**					
**Friendship Quality Score**										
** Companionship and Recreation**	-0.015	0.030	0.265	0.985 (0.929–1.044)	0.606					
** Intimate Exchange**	-0.008	0.023	0.119	0.992 (0.947–1.039)	0.730					
** Conflict and Betrayal**	-0.040	0.029	1.865	0.961 (0.908–1.017)	0.172**					

*p < 0.05

**p < 0.25; AOR: Adjusted odds ratio; COR: Crude odds ratio

## Discussion

Romantic relationship involves the characteristics of intimacy, emotional closeness, and erotic feeling between two individuals [12, 34]. Proxy studies on dating relationship revealed that the prevalence of dating among adolescents in Malaysia was 44.0% [37]. This prevalence is higher than the present study among adolescents in Seremban, Negeri Sembilan, Malaysia. The difference in prevalence may be due to the lack of recent studies on adolescent dating relationship within the Malaysian context, resulting in knowledge gap regarding the comparison of national prevalence.

Besides that, the present study found that 4.6% of the total respondents (adolescents) were in dating relationship. The substantial difference may be attributed to the different definition or criteria used. The present study clearly distinguished dating relationship from romantic relationship, while the former study [37] defined dating relationship in a broad manner, which included both romantic relationship and sexual activity.

Additionally, the recorded prevalence was deemed low among the participating adolescents who reported to have drunk alcohol, have used illegal drugs, and surfed pornography website, as compared to the national prevalence. Studies that were conducted in Norway reported similar findings, where the prevalence of romantic relationship among the students recorded 20.2% [21]. However, the prevalence of adolescent romantic relationship in the US was 32.9%, which appeared to be comparatively higher than that of the present study [18]. This may be due to the cultural differences between the US and Malaysia. Cultural norms in western and eastern countries influence the initiation or formation of romantic relationship differently. Asian-American adolescents were reported to be less likely to have romantic relationship in the past 18 months than Caucasian, African-American, or Hispanic adolescents due to their strict parental supervision [7]. Likewise, Malaysian parents closely supervise their adolescents’ social relationships with opposite gender, contributing to lower prevalence of romantic relationship in adolescence [38].

Meanwhile, studies on the influence of gender on the desire to form romantic relationship have been rather scarce. Generally, female adolescents are less likely to be in romantic relationship compared to male adolescents [18, 39]. In most cases, male adolescents are viewed to develop a relationship with opposite gender for sexual interest, whereas female adolescents are viewed to be more interested in intimacy [40]. Several prior studies demonstrated the significant relationship between male adolescents and sexual activity [37, 41]. Nonetheless, it is common for both male and female adolescents to engage in romantic relationship, as it fulfils adolescence developmental task. However, the engagement in pre-marital sexual activity among adolescents in Malaysia is culturally unacceptable [42]. Most female adolescents are likely to seek romance and stay in romantic relationship without erotic or sexual activity, whereas certain male adolescents are likely to stay in romantic relationship for sexual interest [40]. Based on this perspective alone, it is plausible that why gender may not be an influencing determinant of romantic relationship but a significant determinant of sexual relationship.

The manufacturing of liquor and sale of liquor by premises that exclusively sell liquor were banned during the COVID-19 pandemic, which led to the limited access to purchase alcoholic drinks. Moreover, it is difficult to acquire alcoholic drinks from friends since social gatherings during the country’s national lockdown or movement control order (MCO) during the pandemic were also banned [43]. Thus, all these may have contributed to low prevalence of adolescence alcohol intake. A prior study demonstrated the insignificance of alcohol consumption when controlling for other covariates despite the association between female drinkers and romantic relationships [44]. Another study postulated alcohol as a potential predictor of romantic relationship in certain populations when alcohol consumption is a norm among the students [45].

The current study also found that the participating adolescents who reported using social media to seek romantic relationship were twice more likely to be in romantic relationship than those who did not, which were in line with the findings of another studies in Taiwan and Norway [14, 44]. Unlike the conventional dating means of in-person meeting, an online platform provides easiness in the search for romantic partner, long period of time for mutual communication, and high accessibility to seek and reach out to romantic partner, regardless of location and time. Online platforms, such as online social media (e.g., Facebook) and dating apps (e.g., Tinder), are free from parental supervision and offer unlimited access [46, 47]. These features increase the possibility of adolescent romantic relationship formation. Moreover, the enforcement of MCO limited all social interactions among adolescents and peers physically. Staying at home further increases their mobile phone usage, resulting in higher possibility to interact socially and search for romantic relationship through online platforms.

Moreover, the current study found that the participating adolescents who reported to have surfed pornography website were more likely to be in romantic relationship compared to those who did not. To date, the relationship between pornography website surfing and adolescent romantic relationship has remained underexplored. Unlike reading or browsing pornographic materials through magazines, pornography website surfing through the Internet is highly prevalent, which is contributed by the banning of sales of pornographic magazines, pictures, DVDs, and prints in Malaysia. Pornography website surfing can be accessed via mobile phones or computers anytime. The exposure to online pornography with repeated viewings creates certain ideas of sexual activity with peers and acquaintances and opportunities of erotic activities (e.g., kissing, masturbating, and oral sex), including regular penetrative sexual activities with the partner once a romantic relationship is formed [48]. It may also provide certain ideas of intimacy when a romantic relationship is initiated through the depiction of intimate erotic activities in pornography [49].

Adolescents’ knowledge on healthy romantic relationship has also been very scarcely studied. Numerous prior studies focused on the relationship between SRH knowledge and sexual activity. The current study used SRH knowledge as a proxy to examine adolescents’ knowledge and its relationship with romantic relationship. Poor level of SRH knowledge is related to poor levels of awareness and understanding of the consequences of romantic relationship among adolescents. They tend to form romantic relationship without realising the negative consequences of advancing to sexual relationship stage.

Accordingly, parental divorce affects parent-adolescent relationship. Adolescents seek non-familial relationship to experience the sense of belonging in a group. Divorce may result in low level of parental supervision, as the related relationships become further apart, and single parents are burdened with more workloads and responsibilities at home [50]. As a result, adolescents form closer relationships with their peers and friends. This then increases the probability of them engaging in romantic relationship. In a prior study, the participating adolescents from a secondary school demonstrated maturity and good understanding of divorce [51]. Another recent study revealed that positive coping strategies among Malaysian adolescents helped them to accept divorce circumstances [52]. Divorce causes physical barriers for parents to meet and maintain close relationships with their children. The current technological advancements of smartphones and social media have helped adolescents to improve their relationships with parents when it is difficult to physically meet up.

Evidently, the COVID-19 pandemic negatively affected the mental health of the adolescent population. A study reported the increasing trends of depression prevalence (as high as 57%) among adolescents in six Arab countries, indicating their vulnerability to depression [53]. With the strict enforcement of MCO, quarantine led to the increasing trend of prevalence of depression among Malaysian adolescents. Limited social activities at the time motivated them to interact and carry out leisure activities through online methods, including gaming. The use of the Internet nearly doubled from 5.6 hours per day to 9.7 hours per day among adolescents during the pandemic [53]. A prior study concluded the significant relationships of high Internet use, social media use, and gaming with depression [54]. Apart from that, bullying was reported to cause depression among adolescents [55]. In the current digital era, cyberbullying has become a critical concern given its significant association with depression among adolescents [56]. During the pandemic, all schools, colleges, and universities had to be closed, but daily learning activities progressed online. There are certain challenges like limited access to online learning devices and online learning due to poor Internet connection. As a result, there may be the rising depression trend among adolescents, particularly when they had to deal with tests and examinations.

### Implications

The obtained results and findings of the current study presented several significant implications. Firstly, this study’s results revealed SRH knowledge as the most significant determinant of adolescent romantic relationship within the Malaysian context. Hence, it is recommended for future research to employ the existing SRH modules to conduct a community-based experimental study in order to examine the effectiveness of improving the level of SRH knowledge in regards to adolescent romantic relationship. Secondly, this study’s results demonstrated the need to be aware of the time limit policy for social media use. Thirdly, this study highlighted the importance of targeted health promotion to empower adolescents with knowledge on the consequences, especially adverse health consequences, of pornography website surfing. Fourthly, the high prevalence of depression in this study and its significant association with romantic relationship suggest the need for mass screening and community intervention among adolescents in Malaysia. Lastly, further longitudinal study will be able to investigate the causal relationship between the determinants and romantic relationship.

### Limitations

Despite the significant theoretical contributions of this study on adolescent romantic relationship, there were several limitations to be considered. Firstly, as the current study adopted a cross-sectional design, no temporal relationships of the studied determinants with adolescent romantic relationship were proved. Secondly, cluster random sampling strategy is generally more biased than simple random sampling; as a result, not all adolescents had equal chance of being selected for the study’s questionnaire survey. Thirdly, sample size for this study was calculated according to a proxy study on sexual activity due to the lack of studies on adolescent romantic relationship within the Malaysian context. Fourthly, the use of self-administered questionnaire introduces reporting bias. The current study’s questionnaire included several sensitive questions, which may increase the potential of underreporting. Respondents may indicate “Never” for questions regarding alcohol intake, illegal drug use, or pornography website surfing, as they are concerned that their responses may implicate them in future. In order to minimise reporting bias, respondents were briefed in detail at the start of the questionnaire survey, that every response would be kept strictly private and confidential and would not be disclosed to parents, teachers, or friends. Additionally, recall bias may occur in terms of attempting to remember the parental knowledge on respondents’ location, friends, or friends’ parents during the parental monitoring assessment or the frequency of doing an activity together during the friendship quality score assessment. Besides that, considering the potential of non-response bias, sample size was adjusted to compensate 20% of non-responses. The recorded response rate in this study was found high. Meanwhile, in the case of parental monitoring assessment, parents’ responses were not included in the present study due to time constraints. Therefore, parental monitoring was assessed from the perspectives of adolescents, which may contribute to respondent bias.

## Conclusion

The recorded prevalence of adolescent romantic relationship within the Malaysian context was low compared to a previous study in Malaysia. This study demonstrated the significant role of social media use, pornography website surfing, SRH knowledge, and depression on adolescent romantic relationship. Further longitudinal studies to investigate the temporal relationships are recommended. Intervention programmes which improve the level of SRH knowledge among adolescents and which empower parents with the necessary knowledge and skills to discuss SRH topics with their adolescents are also highly suggested.
